# Genetic diversity and population structure of *Aedes aegypti* after massive vector control for dengue fever prevention in Yunnan border areas

**DOI:** 10.1038/s41598-020-69668-7

**Published:** 2020-07-29

**Authors:** Rui-chen Lv, Chang -qiang Zhu, Chun-hui Wang, Le-le Ai, Heng Lv, Bing Zhang, Chun-min Li, Jing An, Pei-gang Wang, Dan Hu, Xian-zhe Tan, Lu Yang, Hong-ning Zhou, Wei-long Tan

**Affiliations:** 1 Department of Vector Control, Huadong Research Institute for Medicine and Biotechnics, Nanjing, 210002 Jiangsu China; 2 The Key Laboratory of Insect Control of Insect Vectors in Yunnan Province, The Key Technology Innovation Team for Prevention and Control of Insect Vectors in Yunnan Province, Yunnan Institute of Parasitic Diseases, Pu’er, 665000 Yunnan China; 3 Department of Microbiology, School of Basic Medical Science, Capital Medical University, Beijing, 100069 China; 4 Nanjing Foreign Language School, Nanjing, 210008 Jiangsu China

**Keywords:** Computational biology and bioinformatics, Phylogeny, Genetics, Genetic markers, Haplotypes

## Abstract

Dengue fever is a mosquito-borne disease caused by the dengue virus. *Aedes aegypti* (*Ae. Aegypti*) is considered the primary vector of Dengue virus transmission in Yunnan Province, China. With increased urbanization, *Ae. aegypti* populations have significantly increased over the last 20 years. Despite all the efforts that were made for controlling the virus transmission, especially on border areas between Yunnan and Laos, Vietnam, and Myanmar (dengue-endemic areas), the epidemic has not yet been eradicated. Thus, further understanding of the genetic diversity, population structure, and invasive strategies of *Ae. aegypti* populations in the border areas was vital to uncover the vector invasion and distribution dynamic, and essential for controlling the infection. In this study, we analyzed genetic diversity and population structure of eight adult *Ae. Aegypti* populations collected along the border areas of Yunnan Province in 2017 and 2018. Nine nuclear microsatellite loci and mitochondrial DNA (mtDNA) sequences were used to achieve a better understanding of the genetic diversity and population structure. One hundred and fourteen alleles were found in total. The polymorphic information content value, together with the expected heterozygosity (He) and observed heterozygosity (Ho) values showed high genetic diversity in all mosquito populations. The clustering analysis based on Bayesian algorithm, the UPGMA and DAPC analysis revealed that all the eight *Ae. aegypti* populations can be divided into three genetic groups. Based on the mtDNA results, all *Ae. aegypti* individuals were divided into 11 haplotypes. The *Ae. aegypti* populations in the border areas of Yunnan Province presented with high genetic diversity, which might be ascribed to the continuous incursion of *Ae. aegypti*.

## Introduction

Dengue fever (DF) is an acute infectious disease transmitted by *Aedes* mosquitoes and one of the major public health problems worldwide. More than 3.9 billion people across 128 countries are at risk of DF infection, while some 100–400 million infections occur each year^1^. Since the first outbreak of DF in China, which occurred in Guangdong province in 1978, DF has been causing epidemic cycles every 4–7 years in China^2^.

Over recent years, an increase in human cases of DF has been observed in China^3^^,^ especially in the Yunnan Province. This southeast Chinese province comprises 16 prefectures and 129 counties and is characterized by a tropical and subtropical climate, which is conducive to dengue virus infection^4^. Besides, Yunnan Province shares a 4,060-km border with Laos, Vietnam, and Myanmar, all of which are dengue-endemic areas^2^. Since 2004, sporadic imported cases of DF have almost been reported annually in Yunnan Province, China, while the first cases of local infection occurred in 2008 in Dehong prefecture and Lincang City^5,6^. In 2013, a large-scale dengue outbreak occurred in Dehong and Xishuangbanna prefectures.

DF prevention and control depend on effective vector control measures. However, DF has four different serotypes that may affect epidemic control. *Aedes aegypti* and *Ae. Albopictus* mosquitos are the main vectors that transmit the virus to humans^7^. These mosquitoes are widely distributed in tropical regions, especially in Southeast Asia^8^. *Ae. aegypti* is mainly found in densely populated urban areas^9^. In Yunnan province, the first case of *Ae. aegypti*-borne viral infection was reported in Jiegao Port, Ruili, in 2002^10^. At present, the distribution of *Ae. aegypti* is still limited to border port areas and has not spread to the inland area of Yunnan Province. However, it has been gradually adapting the climate and environment of Yunnan Province. It has been confirmed by field monitoring that a large number of breeding sites has been found in Dehong, Longchuan County and other ports in nearby areas, and it has become a new member of mosquito population in this area^4,11–13^.

Shi et al.^14^ indicated a population invasion of *Ae. aegypti* in the border areas of Yunnan Province. Over the last few years, Yunnan Province has taken measures to comprehensively control *Ae. aegypti* populations in this area. For example, the “Patriotic Health Movement” and “Building a civilized city” campaigns were carried out in the border cities. The ports of Yunnan Province adopted integrated prevention and control methods to control the vectors, using environmental governance and vector monitoring so as to cut off the transmission channels and prevent large-scale invasion and spread of vectors in the region. However, the DF epidemic has become a serious public health threat, which kept being among the top 5 reported cases of the infectious diseases in Yunnan Province. Therefore, it is necessary to continue implementing the analysis of *Ae. aegypti* population and the tracking of invasive events.

Genetic techniques could be used to infer the pathways and sources of invasive species, inform the genetic composition and demographic history of founding populations^15,16^. Microsatellites, also known as simple sequence repeats (SRSs) or short tandem repeats (STRs) are short, tandemly repeated DNA motifs of 1–6 nucleotides distributed throughout eukaryotic genomes^17^. The microsatellite markers show the advantages of high polymorphism, co-dominant expression, and extensive genomic distribution, and have been proven useful for detecting the subtle population structure^18^. Mitochondrial DNA (mtDNA) is commonly used in molecular evolution studies of insects^19^. The mtDNA is more sensitive to genetic drift and has greater genetic differentiation. It is particularly useful for detecting genetic differences and reconstructing the transmission history of invading mosquitoes^20,21^. Previously, nuclear microsatellite markers and mtDNA sequences were used to investigate the genetic diversity and population structure of *Ae. aegypti* across the Yunnan Province region^14,22^. The whole genetic information can inform us on the invasion routes, the colonizing capacity, adaptability, and behaviors of invading mosquito lineages^23^.

In this study, we analyzed genetic diversity and population structure in eight populations of adult *Ae. Aegypti* those were collected along the border area of Yunnan Province in 2017 and 2018.

## Results

### Microsatellite genetic diversity

The polymorphic information content (PIC) value is one of the indicators used to measure allele richness of genes. A total of 114 alleles were found for the nine genetic markers; all alleles were sequenced. As shown in Table 1, the microsatellite locus SQM 6 had the highest number (20) of alleles, while marker SQM 1 had the lowest number (7) of alleles. The PIC values were high, ranging from 0.392 to 0.886, and the average value was 0.672, which indicated that sites were highly polymorphic and can reflect the genetic characteristics of all *Ae. aegypti* populations^24^. The microsatellite locus SQM 6 had the highest PIC value (0.886), whereas the locus SQM 1 the lowest PIC value (0.392) (Table 1).

**Table 1:** The PIC values and the number of alleles of all *Ae. aegypti* samples.

Locus	Number of alleles	PIC
SQM 1	**7**	**0.392**
SQM 2	16	0.804
SQM 3	13	0.629
SQM 4	12	0.633
SQM 5	9	0.679
SQM 6	**20**	**0.886**
SQM 7	10	0.657
SQM 8	11	0.614
SQM 9	16	0.755
Mean	13	0.672

Bold values indicates the largest number of alleles and the largest PIC value.

**Figure 1:**
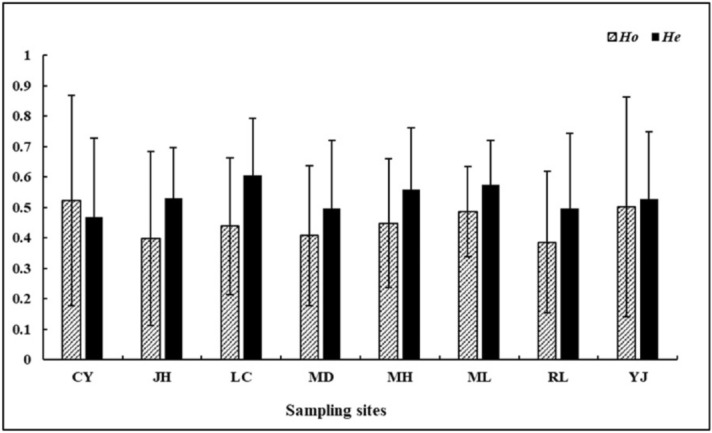
The Ho and He values of all *Ae. aegypti* populations.

Normally, the genetic diversity of the mosquito population is positively related to the expected heterozygosity (He) and observed heterozygosity (Ho) value of the population. *He* and *Ho* value of all *Ae. aegypti* populations ranged from 0.385 to 0.605, which suggests that the genetic diversity of all mosquito populations is relatively high (Fig. 1). Except for CY, the *Ho* value in other regions is lower than the *He* value, which indicates that there may be inbreeding within the species. In addition, CY may have a large amount of individual external supplements or may experience bottlenecks.

Nearly all the F_IS_ values in all *Ae. aegypti* populations, except population YJ, were positive, ranging from 0.05882 to 0.24657 (Table 2), which further indicated that these populations contained different degrees of inbreeding and Heterozygote deficiency that also may be the reason for the deviation of all populations from HWE.

**Table 2:** The F_IS_ value of all *Ae. aegypti* populations.

Sampling sites	Sample size	F_IS_ value
YJ	9	− 0.09422
LC	11	0.22550
ML	15	0.24492
RL	15	0.24560
JH	15	0.16949
MD	15	0.21845
MH	15	0.24657
CY	10	0.05882

### Microsatellite genetic structure

The F_IT_ value over all populations was 0.309, which indicated that there were significant population differences among the individuals screened. The AMOVA results indicated that the largest proportion of genetic variation in *Ae. aegypti* population existed in individuals and individuals within populations, accounting for 69.14% and 15.69% of the variation, respectively (Table 3). Although the sampling site is an important factor (*P* < 0.0001), the ratio was relatively low (Table 3). The Bottleneck effect analysis of all populations revealed that nearly all populations, except population ML and RL, are mutation-drift equilibrium (Table 4).

**Table 3:** Hierarchical analysis (AMOVA) of the genetic variation in the *Ae. aegypti* samples.

Source of variation	*df*	x^2^	Variance component	Variation %	*P *value	Fixation index
Among sites	2	32.066	0.13994 Va	5.6	0.01188	F_CT_ = 0.05597
Among populations within sites	5	45.426	0.23947 Vb	9.58	< 0.0001	F_SC_ = 0.10146
Among individuals within populations	97	243.761	0.39221 Vc	15.69	< 0.0001	F_IS_ = 0.18494
Within individuals	105	181.5	1.72857 Vd	69.14	< 0.0001	F_SC_ = 0.30863

**Table 4:** The Bottleneck effect analysis of all *Ae. aegypti* populations.

		CY	JH	LC	MD	MH	ML	RL	YJ
SMM	*He* < *Heq*	4	5	3	4	5	7	8	2
	*He* > *Heq*	4	4	6	4	4	2	1	6
	P(*He* < *Heq*)	0.43875	0.28140	0.44234	0.43941	0.30623	**0.02471**	**0.00566**	0.27417

Bold values indicates significant differences

**Figure 2:**
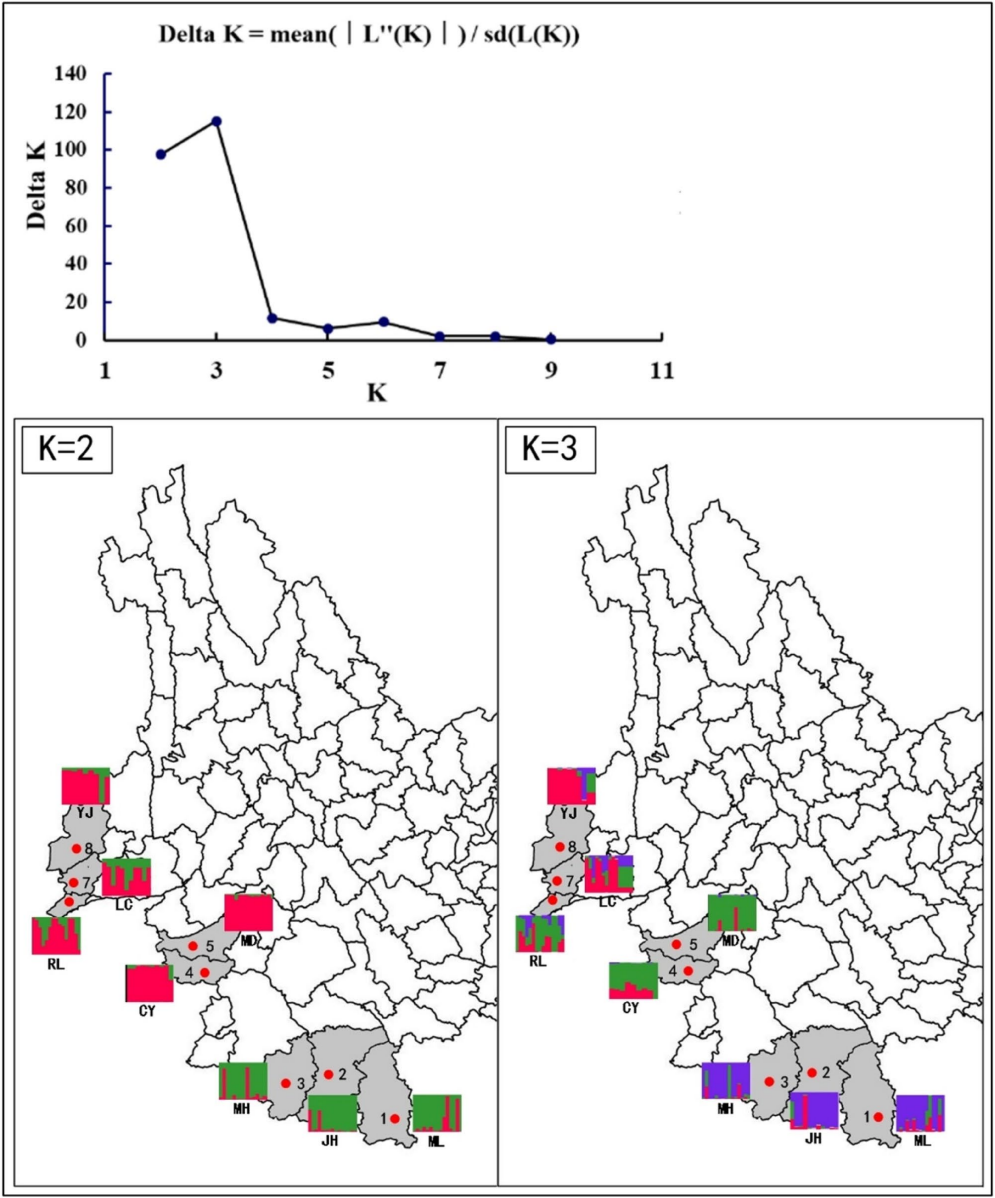
The clustering analysis of all *Ae. aegypti* populations based on the Bayesian algorithm. Structure bar plot for all *Ae. aegypti* populations used in this study. The height of each color represents the probability of assignment to a specific cluster. Subdivision of all the individuals into K = 2 and K = 3 clusters.

**Figure 3:**
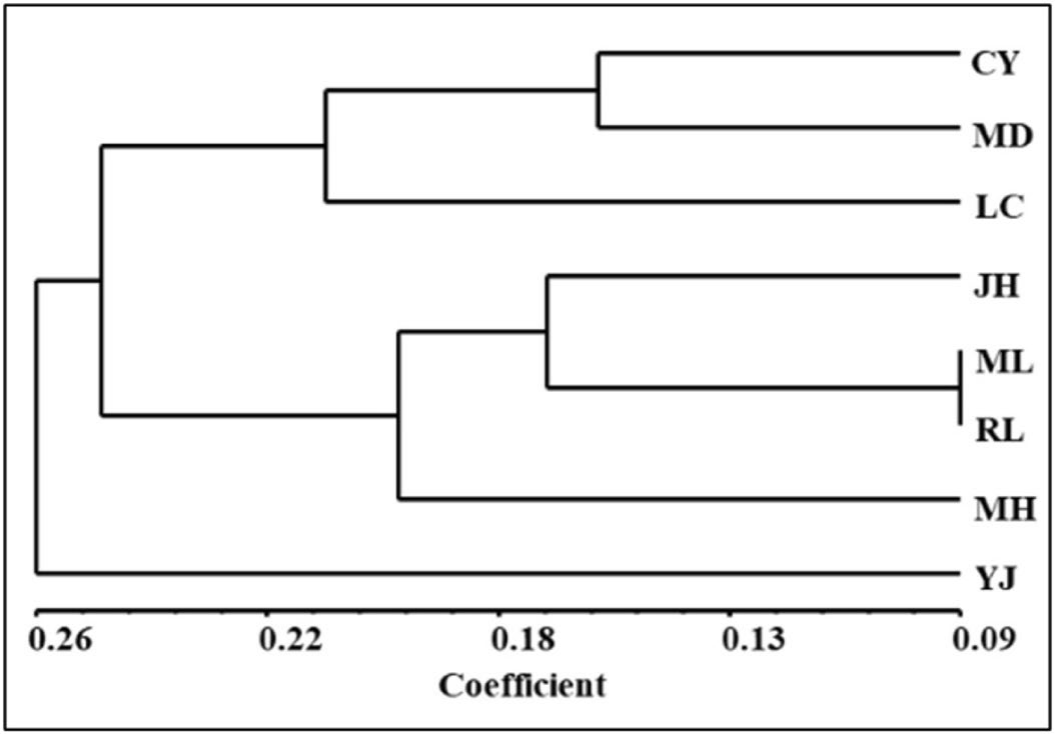
The UPGMA analysis of all *Ae. aegypti* populations. UPGMA cluster analysis of 8 sampling locations based on the genetic distance, the evolutionary distances were computed using the maximum composite likelihood method.

**Figure 4:**
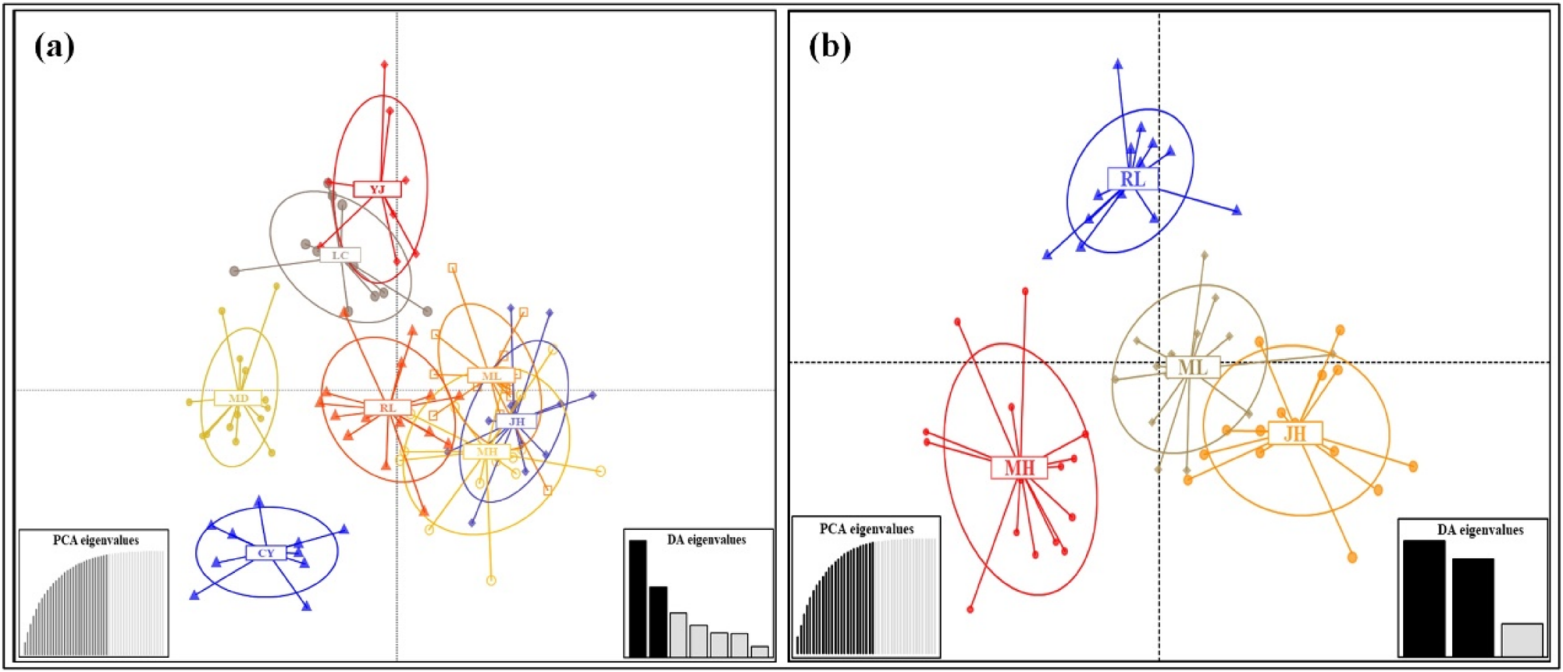
Population structure described by discriminant analysis of principal components (DAPC) based on nine nuclear microsatellite loci. Each color corresponds to a single population, and ellipses with dashed lines represent individuals. (**a**) All eight populations of *Ae. aegypti* (a total of 96.6% of the variation was explained by 30 PCs in the DAPC analysis); (**b**) Four closely related populations of *Ae. aegypti* (a total of 96.4% of the variation was explained by 25 PCs in the DAPC analysis).

In order to analyze the specific genetic structure of all eight *Ae. aegypti* populations, based on the Bayesian algorithm, a clustering analysis was carried out (Fig. 2). Combining with The UPGMA and DAPC analyses, revealed that all eight *Ae. aegypti* populations can be divided into three genetic groups of the populations from Lincang city, Xishuangbanna prefecture and Dehong prefecture were genetically correlated, except for RL population, which was highly related to the population Xishuangbanna prefecture (Figs. 3, 4).

**Figure 5:**
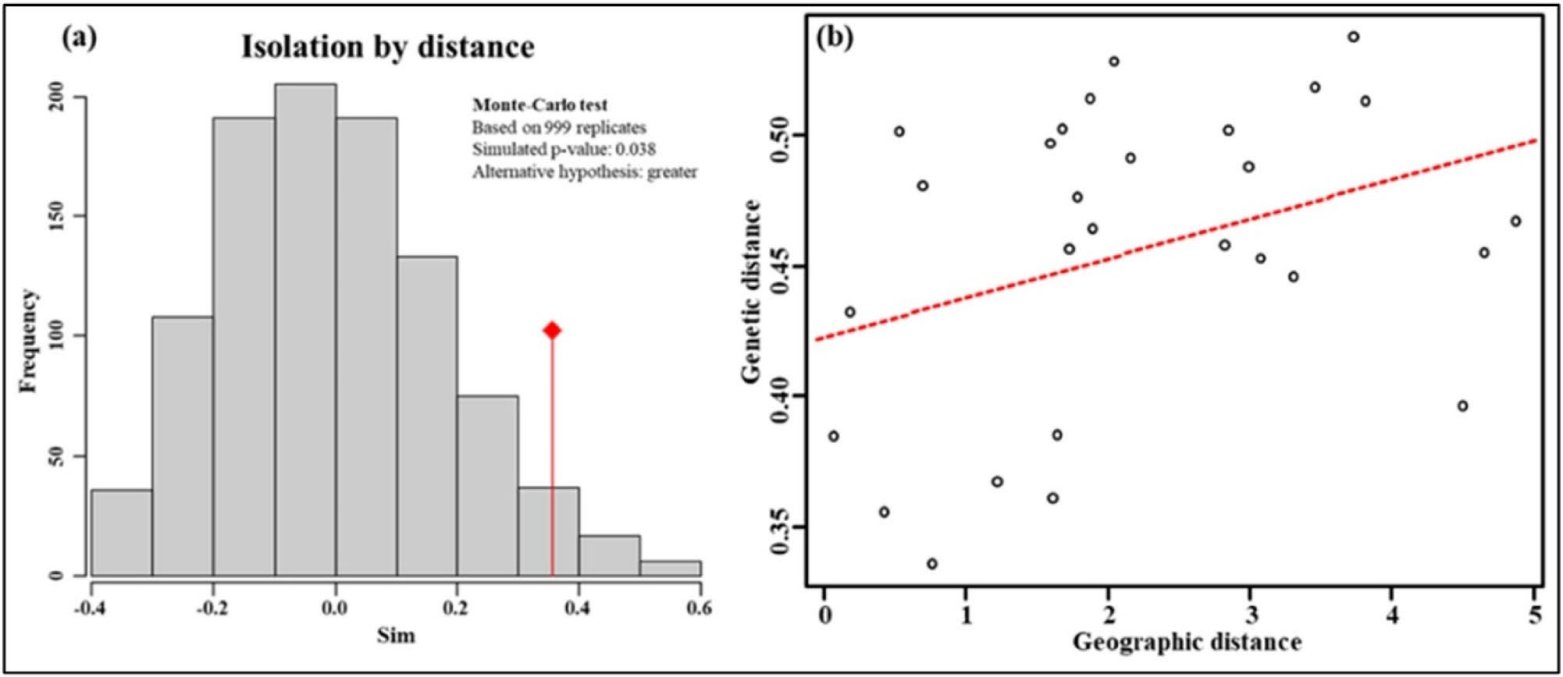
The isolation by distance analysis results among all eight *Ae. aegypti* populations. (**a**) The original value of the correlation between the distance matrices is represented by the red dot, while histograms represent permuted values (i.e., under the absence of spatial structure). The original value being out of the reference distribution represents the significant spatial structure. (**b**) The red line represents the positive relationship between genetic distance and geographic distance among all individuals.

The IBD analysis displayed that the genetic distance of all eight *Ae. aegypti* populations were positively related to geographic distance, which meant the geographical isolation was the primary cause of genetic diversity of *Ae. aegypti* (Fig. 5). While the *Ae. aegypti* can only move hundreds of meters around their larval habitats, which suggests that the transmission of *Ae. aegypti* in Yunnan Province does not depend on its own activities, but on other factors, such as human activities.

The pairwise F_ST_ values of *Ae. aegypti* ranged from 0.061 to 0.220 (Table 5), showing significant genetic differences. All *P *values were significant (*P* < 0.05) after Bonferroni corrections were applied.

**Table 5:** Pairwise population differentiation estimates (F_ST_) (below the diagonal) and geographical distance [ln(km)] (above the diagonal) between all populations of *Ae. aegypti*.

	CY	JH	LC	MD	MH	ML	RL	YJ
CY	0.000	1.598	1.790	0.427	1.683	2.859	1.648	2.051
JH	**0.191**	0.000	3.083	2.164	0.071	0.764	2.828	3.818
LC	**0.072**	**0.105**	0.000	1.727	3.463	4.649	0.187	0.536
MD	**0.107**	**0.220**	**0.140**	0.000	1.897	2.999	1.620	1.879
MH	**0.153**	**0.085**	**0.155**	**0.164**	0.000	1.222	3.311	3.730
ML	**0.156**	**0.061**	**0.073**	**0.195**	**0.074**	0.000	4.504	4.873
RL	**0.120**	**0.163**	**0.080**	**0.138**	**0.129**	**0.062**	0.000	0.705
YJ	**0.179**	**0.174**	**0.101**	**0.210**	**0.163**	**0.121**	**0.158**	0.000

The significances were tested for multi comparisons by the Bonferroni method, *P* < 0.05; bolding displayed below the diagonal means significant difference; F_ST_ averaging between all populations displayed below the diagonal; geographical distance [ln(km)] displayed above the diagonal.

### Haplotype networks and diversity

**Figure 6:**
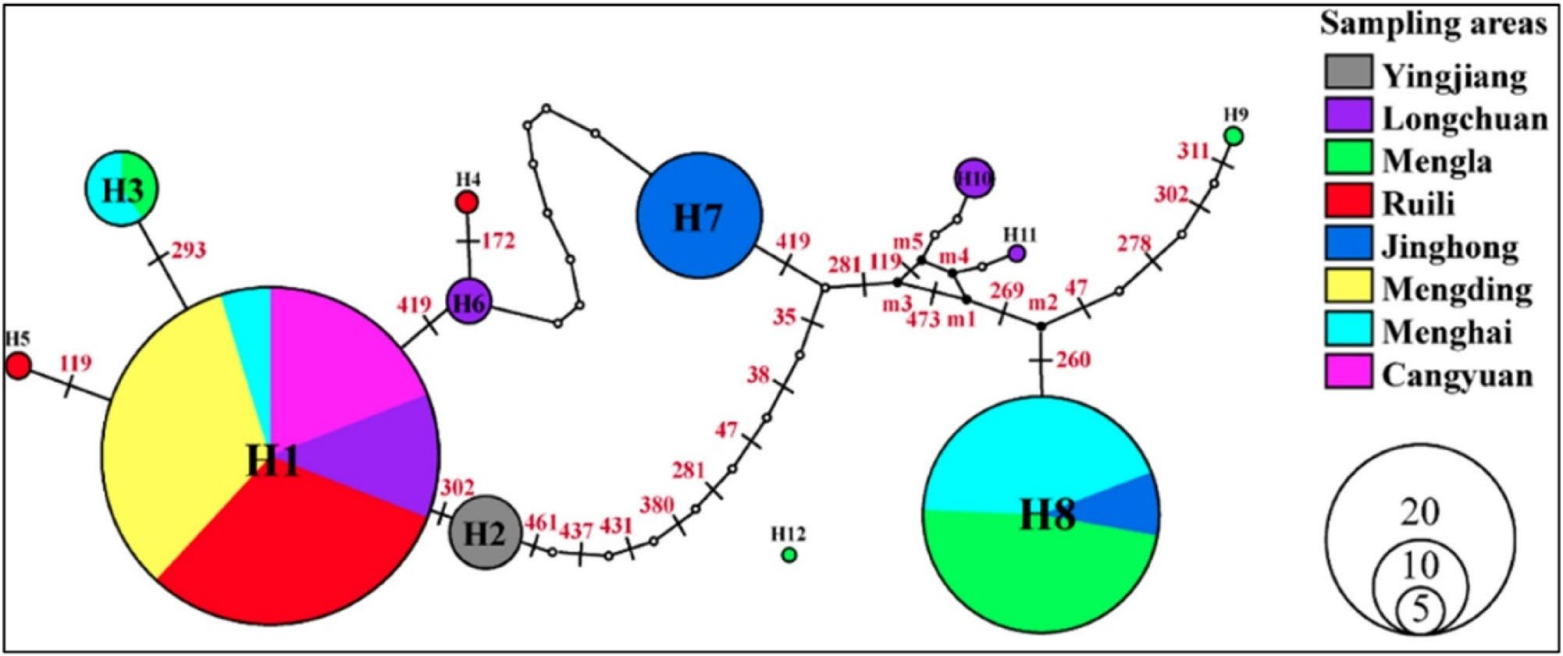
The Haplotype analysis of all *Ae. aegypti* populations based on mitochondria COI and ND4. The network of haplotypes. Each nonsolid black colored circle represents observed haplotype with greater circle size indicating a greater number of individuals with that haplotype. The black line with numbers represents the position of mutant bases. The colors correspond to the different sampling areas.

**Figure 7:**
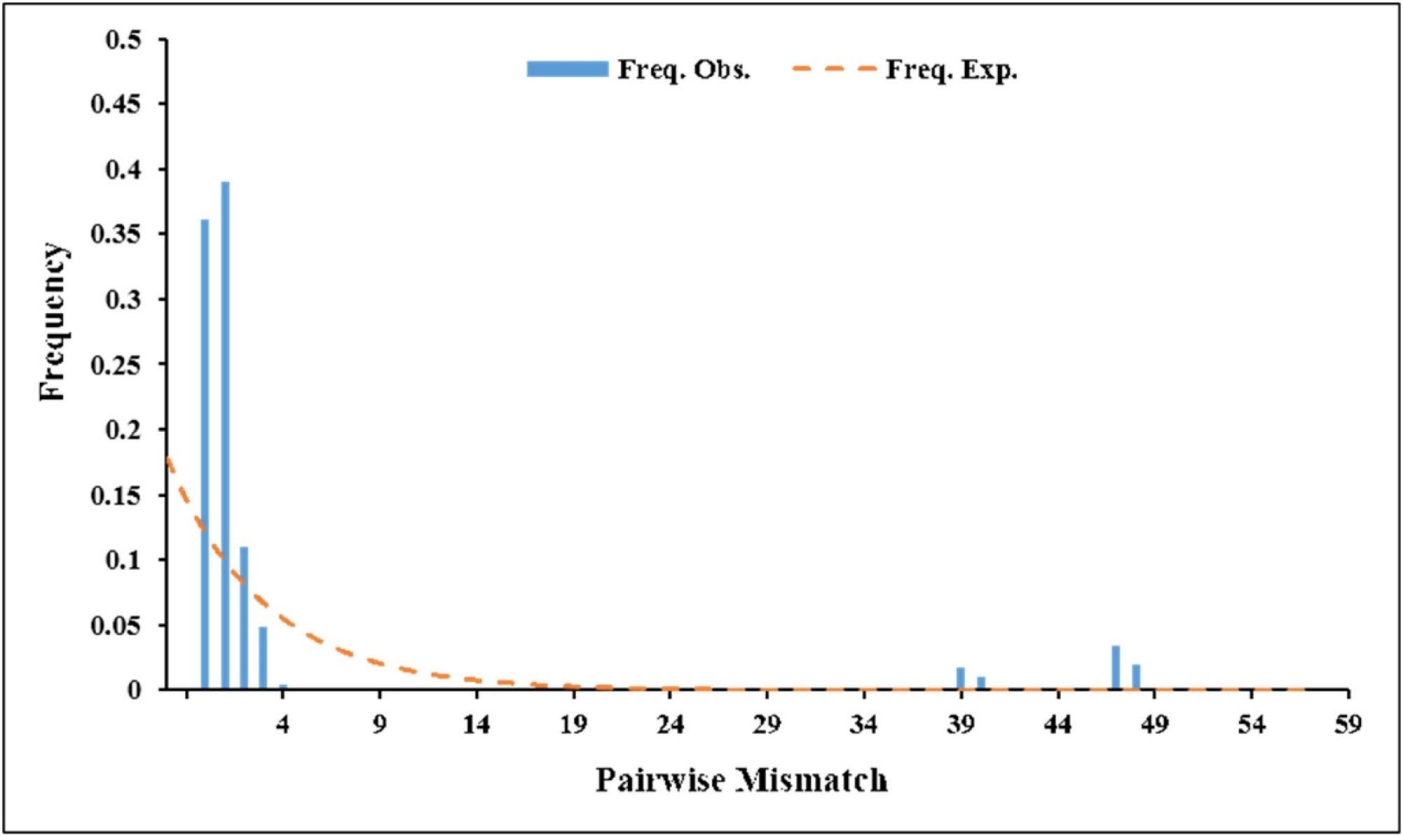
The Neutral test and mismatch analysis of all *Ae. aegypti* populations based on mitochondria COI and ND4. The mismatch distributions showed a smooth and main unimodal curve peaks, which coincide with the population expansion model.

Based on the mitochondrial COI, ND4, and ND5 areas of *Ae. aegypti*, all *Ae. aegypti* individuals from nine populations were divided into 11 haplotypes, among which H1, 2, 3, 7, and 8 were the main haplotypes. The distribution of these haplotypes in a specific population is shown in Fig. 6. All the new sequences generated in this study are available from GenBank (Table 6). Among the eleven haplotypes, H1, 2, 3, 7, and 8 were the main haplotypes in all populations. The haplotype H1 had the most individuals, mostly from MD, MH, CY, LC, and RL, while H3 and H8 were mainly distributed at Xishuangbanna prefecture and H2 and H7 were only distributed at YJ and JH, respectively (Fig. 6). The vast majority of individuals have shared haplotypes, and sampling sites had individuals that belonged to haplotypes. Neutral test and mismatch analysis based on the mitochondrial genes ND4 and ND5 of *Aedes aegypti* showed that the distribution of nucleotide mismatches in this population had a single peak structure, indicating that the population of *Ae. aegypti* had experienced at least one significant population expansion (Fig. 7).

**Table 6:** Sequenced information for all 8 *Ae. aegypti* populations from Yunnan province, China.

Number	Haplotype code	GenBank number
1	H01	MT621022
2	H02	MT621023
3	H03	MT621024
4	H04	MT621025
5	H05	MT621026
6	H06	MT621027
7	H07	MT621028
8	H08	MT621029
9	H09	MT621030
10	H10	MT621031
11	H11	MT621032
12	H12	MT621033

## Discussion

The information on invasion and spread of mosquito vectors is essential for understanding vector-borne disease outbreak, transmission dynamics among human populations and implementing effective mosquito control programs^25^. These are all important factors influencing the mosquito population dynamics, genetic structure patterns, and pathogen transfer through vector populations^26^. *Ae. aegypti* is the most important epidemic vector that can cause DF and dengue hemorrhagic fever (DHF) in human and is mainly distributed in southeast China. The most suitable habitats of *Ae. aegypti* include Hainan, Guangdong, Guangxi, the western and southern border areas of Yunnan, and parts of the southern Guizhou region^27^. Yet, due to climate changes and increased urbanization, a significant northward shift occurred in the northern Chinese region over recent years^28^.

*Ae. aegypti* is an invasive species and potential vector of disease agents in China, which has a significant impact on public health. *Ae. aegypti*-associated infection was first reported in Yunnan Province (Jiegao Port, near Ruili City, Dehong prefecture) in 2002^29^. In 2009, the *Ae. aegypti* was detected for the first time in Guanlei Port, Mengla city, Xishuangbanna prefecture^30^, and later on (2014) in Lincang, Mengding county^31^. The distribution range and abundance of *Ae. aegypti* species have significantly increased, and were established in at least eight cities in Yunnan Province. Therefore, the monitoring of *Ae. aegypti* species is essential for preventing and controlling vector-borne infectious diseases. In our study, all the *Ae. Aegypti* samples were collected from eight sampling places in three prefectures of Yunnan Province (Xishuangbanna prefecture, Lincang city, and Dehong prefecture). The DF cases in Yunnan Province mainly originated from these prefectures.

Our population genetics analyses of the populations from Yunnan border area that were based on two types of genetic markers (Microsatellites and mtDNA) revealed the genetic structure and the population distribution within this region. The PIC value, He and Ho value were important parameters for measuring the genetic diversity of a population; the higher the value, the more complex the population structure is. Our results revealed that *Ae. aegypti* species in the Yunnan border region had a great allelic variation. The *Ae. aegypti* mosquitoes may easily transmit the virus to humans and usually find shelter in indoor habitats. Their flight range is limited, which means they can only move hundreds of meters around their larval habitats^25,32^. The relatively high genetic diversity of all mosquito populations is most likely caused by invasion events and human activities^33^. The results of IBD analysis support this conclusion that the dispersal of *Ae. aegypti* species is aided by human activities and transportation in Yunnan Province.

Bayesian algorithm-based population analysis showed that all *Ae. aegypti* populations could be divided into three genetic groups. The first group had four populations in the Xishuangbanna prefecture (JH, MH, and ML) and RL city, which might be related to the close tourism and commercial trade exchanges between these two regions. The second group represented two populations from Lincang City. The third group was composed of LC and YJ. Contrary to other six populations, YJ population is closely related with LC population, which is significantly different from the UPGMA result. The differences may come from the sample size and range.

Except for Yingjiang, the inbreeding coefficient (F_IS_) values of eight other *Ae. aegypti* populations were positive. Combined with the UPGMA and DAPC analysis, the results indicated that there may have a recent invasion and colonization of the *Ae. aegypti* in YJ city. Due to the limited flight range, this phenomenon is common for *Ae. aegypti* population on a small spatial scale^33^. In 2016, the Xishuangbanna Prefecture established a “Spring Patriotic Health Movement” with the scope to provide the integrated control for infectious disease vectors. This may explain the bottleneck effect observed in ML and RL.

MtDNA markers have been widely used to evaluate the genetic diversity of *Ae. aegypti* populations^34,35^. In our study, the degree of polymorphism found in the COI and ND4 sequences were relatively high (eight populations were divided into eleven haplotypes). The H1, which was the dominant haplotype, was found in five places. The analysis of all mosquito samples from two localities in Lincang City (MD and CY) showed only one haplotype (H1) for each gene. Dehong Prefecture is close to Myanmar border, and the intensive personnel activities have led to a large number of invasion events. The abundant waters and commercial activities in Xishuangbanna prefecture have also contributed to many invasion events. This idea was supported by the high levels of polymorphism detected in Xishuangbanna and Dehong prefectures (six haplotypes and seven haplotypes, respectively), which may be the main entry points of *Ae. aegypti* in Yunnan Province. The H2 haplotype was only distributed at YJ independent from other regions. Combined with the negative F_IS_ value of population YJ, the *Ae. aegypti* species likely invaded Yunnan Province from this region over recent years.

Our research shows that in Dehong and Xishuangbanna prefectures, *Ae. aegypti* population invade these areas because of the continuous tourist and business activities. Inspection and quarantine need to be strengthened at the border ports and further investigation and research on mosquito vectors should be carried out. The government needs to designate effective prevention and control measures, strengthen environmental governance in the border areas and implement mosquito control measures.

## Conclusion

The nuclear microsatellite markers and mtDNA sequences (COI, ND4, and ND5) were used to uncover the population genetics of the *Ae. aegypti* in the border area of Yunnan Province. Although several attempts have been made by the government of Yunnan Province to control the mosquito vectors, the *Ae. aegypti* populations in this region showed high genetic diversity and genetic structure due to the continuous invasion, and increased urbanization. Our research confirms that, over recent years, a significant *Ae. aegypti* invasive event occurred in YJ City; and that the Xishuangbanna and Dehong prefectures were important areas for the *Ae. aegypti* invasion.

In summary, our results suggest that the control of *Ae. aegypti* in Yunnan Province is still a demanding task that needs to be taken seriously. Thus, monitoring of suspected cases of DF and the vectors should be enhanced.

## Materials and methods

### Mosquito sampling and DNA isolation

**Figure 8:**
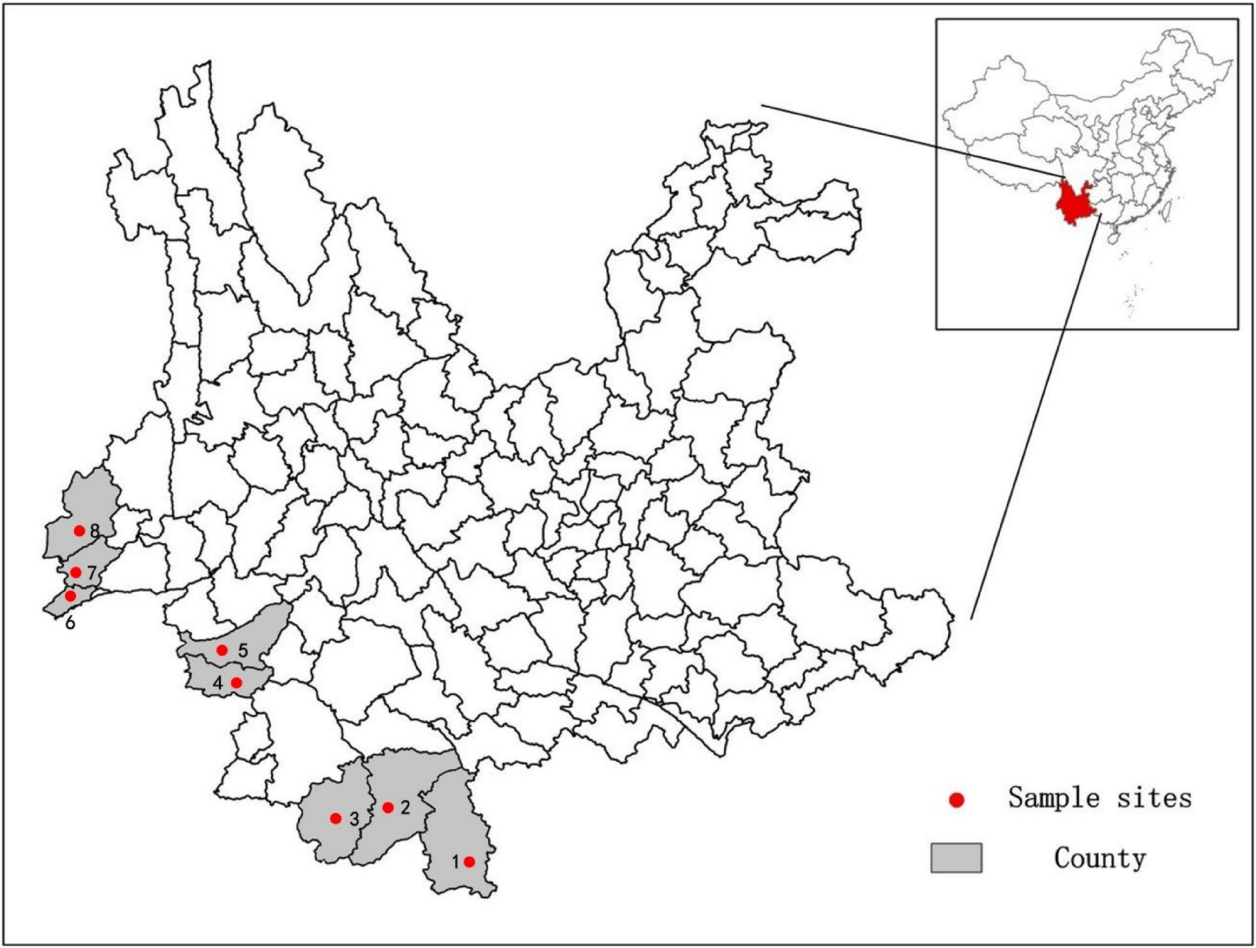
The geographical location of collection sites of *Ae. aegypti* specimens. 1: Mengla (ML); 2: Jinghong (JH); 3: Menghai (MH); 4: Cangyuan (CY); 5: Mengding (MD); 6: Ruili (RL); 7: Longchuan (LC); 8 Yingjiang (YJ).

All the adult *Ae. aegypti* samples were collected from following eight locations along the border area of Yunnan Province between May 2017 and September 2018 (Fig. 8, Table 7). Each collection site covered an area of approximately 500 m in diameter. According to the Surveillance Methods for Vector Density-Mosquito (GB/T 23797–2009), a hand-held aspirator was used to collect the adult mosquitoes (intercepted before biting). All the samples were identified through the analysis of morphological characteristics in the wild field^36^ and preserved in 100% ethanol at 4 °C for the isolation of genomic DNA^37^.

**Table 7:** Sampling information of *Ae. aegypti* collection in Yunnan Province, China.

Number	Collection region	Location name (code)	Coordination		No. of samples
			Longitude	Latitude	
1	Xishuangbanna	Mengla (ML)	101.57	21.48	15
2		Jinghong (JH)	100.80	22.02	15
3		Menghai (MH)	100.45	21.97	15
4	Lincang	Cangyuan (CY)	99.25	23.15	10
5		Mengding (MD)	99.40	23.55	15
6	Dehong	Ruili (RL)	97.85	24.02	15
7		Longchuan (LC)	97.80	24.20	11
8		Yingjiang (YJ)	97.93	24.72	9

According to the standard DNA extraction procedure, genomic DNA was isolated from individual mosquito sample with the TaKaRa Mini-BEST Universal Genomic DNA Extraction Kit (Takara, Dalian, China); the quality and quantity of extracted DNA were analyzed using NANOdrop1000, after which samples were stored at − 20 °C until further analysis.

### PCR amplification and microsatellite genotyping

Nine microsatellite polymorphic loci were screened from 58 loci, which were described in previous studies by denaturing polyacrylamide gel electrophoresis^38,39^. The primer sequences and information are summarized in Table 8^,^ forward primers were labeled with a fluorescent dye (FAM, HEX, or TAMRA). All the samples were amplified in the 25 μL reaction, which consisted of 2.5 μL 10 × PCR Buffer, 1 μL 1:10 DNA template, each primer at 0.3 μM, dNTPs at 0.25 μM, MgCl_2_ at 1.5 mM, TaqDNA polymerase 1.25U and ddH_2_O. The PCR conditions were as follows: 94 °C for 5 min; 35 cycles at 94 °C for 45 s (for each locus, different annealing temperature was used (Table 8) for 45 s) and 72 °C for 45 s. The final extension was performed at 72 °C for 10 min.

**Table 8:** Primer information of nine microsatellite loci.

Locus	Primer sequence (5′–3′)	Repeat motif	Allele size (bp)
SQM 1	F: AATCGTGACGCGTCTTTTG	CT10(TT)CT	233–239
	R: TAACTGCATCGAGGGAAACC		
SQM 2	F: CAAACAACGAACTGCTCACG	GA15	157–183
	R: TCGCAATTTCAACAGGTAGG		
SQM 3	F: ATTGGCGTGAGAACATTTTG	CAT7	156–186
	R: GAGGAGTGAGCAGATAGGAGTG		
SQM 4	F: GCCAAAAACCAACAAACAGG	TAGA8	286–290
	R: AATCGACCCGACCAATAACA		
SQM 5	F: GGAGCATTCATAGAGAATTGTCA	ATA36	110–116
	R: GAGATGAACCAGTCATAGGGC		
SQM 6	F: CGACAGATGGTTACGGACGG	(TTTA)7(T)14	228
	R: GTCCCGCTCCAAAAATGCCC		
SQM 7	F: AAAACCTGCGCAACAATCAT	AG4	147–169
	R: AAGGACTCCGTATAATCGCAAC		
SQM 8	F: TGATCTTGAGAAGGCATCCA	AG5	170–180
	R: CGTTATCCTTTCATCACTTGTTTG		
SQM 9	F: TCCGGTGGGTTAAGGATAGA	AC1	193–209
	R: ACTTCACGCTCCAGCAATCT		

All PCR amplification products were verified by electrophoresis of 3 μL on a 1.5% agarose gel. The formamide was mixed with LIZ 500-labeled size standard using a ratio of 100:1, and 15 μL mixture was added into the sample plate. The PCR amplification products were diluted at 1:10, 1 μL was added into the reaction, and then run on an ABI3730XL (Applied Biosystems, Foster City, USA) capillary sequencer. All microsatellite alleles were evaluated using GeneMapper software (Applied Biosystems)^14^.

### Microsatellite data analysis

#### Genetic diversity

The PIC values of all nine loci were calculated with PIC-Calc 0.6^14^. The genetic diversity of all *Ae. Aegypti* populations were characterized by expected heterozygosity (*He*) and observed heterozygosity (*Ho*), using POPGENE version 1.32. The F_IS_ value of each mosquito population was also calculated. The statistical significance test was performed with the exact tests available in POPGENE^40^.

#### Genetic structure

The genetic variation was tested by the AMOVA test with Arlequin (version 3.5.2.2) for the interpretation of genetic variability and structure among different locations, mosquito populations. The AMOVA was evaluated at four different hierarchical levels: (1) all samples (non-grouped) were analyzed as a single group to test the overall genetic differences between samples; (2) the samples in one region were analyzed as a unique group; (3) the interregional populations were analyzed as an unique group; (4) the individual sample within population was analyzed as an unique group. Based on the stepwise mutation model (SMM), the recent genetic bottleneck in each mosquito populations was calculated by the software BOTTLENECK 1.2.02. The data were analyzed with the recommended settings: an index statistic closer to 1 indicates that the population is in a stable state, while a very low value indicates that the population has experienced a genetic bottleneck in the past^41^. The sign test implemented in the software was used to test for significant heterozygosis excess. The isolation by distance (IBD) was estimated with Mantel's test in R, using the correlation between genetic distance and geographic distances by the regression of pairwise FST/(1 − FST) on the natural logarithm (Ln) of straight-line geographic distance.

For the determination of real genetic clusters (K) within all mosquito samples, a Bayesian clustering algorithm-based software STRUCTURE 2.2 was employed. All mosquitos were divided into different populations represented by a specific number (K = 8), under the assumption of Hardy–Weinberg equilibrium and linkage equilibrium^42^. The software parameters were set as follows: the assumed populations ranged from 1 to 8, and the calculation model was set as admixture ancestry and independent allele frequency models 100,000 burn-in steps followed by 1,000,000 MCMC replicates, and each population was calculated for 10 runs. The optimum K value was estimated with Evanno’s △k method based on the second-order rate of change in the log probability of the △k among 10 runs of each assumed K^43^, and all the results were uploaded to a web-based utility Harvest for the calculation of the optimum K value (https://taylor0.biology.ucla.edu/struct_harvest/). Furthermore, the software CLUMPP 1.1.1 and DISTRUCT 1.1 (Rosenberg 2004 and 2007) were also used to calculate the average coefficients of membership across the 10 replicates of real K value, and the final results were displayed. In order to further explore the genetic structure, UPGMA trees were computed with NTsys, based on microsatellite Nei’s genetic distance. The DAPC analyses were conducted under the R (vision 3.6.2) condition with the R package “adegenet 2.1.0”.

### mtDNA analysis and PCR amplification

The mitochondrial genes ND4 and COI of *Ae. aegypti* were chosen as the examination sites for exploring sequence polymorphism. At least ten mosquito individuals from each population were selected from the previous microsatellite analysis and identified by the amplification and sequencing of the COI gene. All the mosquito DNAs were amplified with the ND4 primers, which were composed of ND4 forward-(5′-TGATTGCCTAAGGCTCATGT-3′) and ND4 reverse-(5′-TTCGGCTTCCTAGTCGTTCAT-3′) primers targeting 344 bp fragment. The following PCR procedure was used: 5 min of initial denaturation (94 °C) followed by thirty cycles of 94 °C, 40 s of denaturation at 56 °C, 40 s of annealing at 72 °C, 1 min of extension and a final extension at 72 °C for 5 min. The 50 μL PCR solution was composed by 5 μL 10 × PCR buffer, 0.3 μL TaqDNA polymerase (5 U/L), 5 μL dNTP (2 mmoL/L), 2 μL of each of the forward and reverse primers (10 μM), 5 μL template DNA and ddH_2_O. The PCR amplification products were confirmed by agarose gel electrophoresis. All the PCR products were purified with the PCR Purification Kit (OMEGA D6492-02) and cloned to the pMD18-T vector (TaKaRa, Japan) for the final sequencing with ABI 3730XL automatic sequencer (Applied Biosystem).

#### mtDNA data analysis

All the sequences were analyzed with the software Sequencer 5.0 and BioEdit v7.0.570, and aligned with Clustal W. For the ND4 gene, haplotype diversity (Hd), nucleotide diversity (π) and the Tajima and Fu and Li neutrality tests of all mosquito populations were computed by DNA Sequence Polymorphism v6.12.01. A haplotype network of all mosquito populations was also constructed with the software NETWORK 5.0.1.1 for inferring the relationships of all haplotypes and the distribution of all haplotypes at different locations.

### Ethics approval and consent to participate

Pre-permission (May 2017 to September 2018) was granted for adult mosquito observation, adult mosquito collection, and studies in Yunnan province. All the studies were authorized by the Committee for Animal Welfare and Animal Ethics in the Institute of Parasitic Diseases of Yunnan province, China (address: Yunnan province, People’s Republic of China).

## Abbreviations

AMOVA: Analysis of molecular variance
CDC: Centers for Disease Control and Prevention
DF: Dengue fever
DHF: Dengue hemorrhagic fever
F_IS_: Inbreeding coefficient
Hd: Haplotype diversity
He: Expected heterozygosity
Ho: Observed heterozygosity
IBD: Isolation by distance
mtDNA: Mitochondrial DNA
PCR: Polymerase chain reaction
PIC: Polymorphic information content
SMM: Stepwise mutation model
SRSs: Simple sequence repeats
STRs: Short tandem repeats
WHO: World Health Organization
